# Artificial intelligence in atherosclerotic disease: Applications and trends

**DOI:** 10.3389/fcvm.2022.949454

**Published:** 2023-01-19

**Authors:** Polydoros N. Kampaktsis, Maria Emfietzoglou, Aamna Al Shehhi, Nikolina-Alexia Fasoula, Constantinos Bakogiannis, Dimitrios Mouselimis, Anastasios Tsarouchas, Vassilios P. Vassilikos, Michael Kallmayer, Hans-Henning Eckstein, Leontios Hadjileontiadis, Angelos Karlas

**Affiliations:** ^1^Division of Cardiology, Columbia University Irving Medical Center, New York, NY, United States; ^2^Heart Centre, John Radcliffe Hospital, Oxford University Hospitals, NHS Foundation Trust, Oxford, United Kingdom; ^3^Department of Biomedical Engineering, Khalifa University of Science and Technology, Abu Dhabi, United Arab Emirates; ^4^Institute of Biological and Medical Imaging, Helmholtz Zentrum München, Neuherberg, Germany; ^5^School of Medicine, Chair of Biological Imaging at the Central Institute for Translational Cancer Research (TranslaTUM), Technical University of Munich, Munich, Germany; ^6^Third Department of Cardiology, Hippokration University Hospital, Aristotle University of Thessaloniki, Thessaloniki, Greece; ^7^Department for Vascular and Endovascular Surgery, Klinikum Rechts der Isar, Technical University of Munich, Munich, Germany; ^8^DZHK (German Centre for Cardiovascular Research), Partner Site Munich Heart Alliance, Munich, Germany; ^9^Healthcare Innovation Center, Khalifa University of Science and Technology, Abu Dhabi, United Arab Emirates; ^10^Department of Electrical and Computer Engineering, Aristotle University of Thessaloniki, Thessaloniki, Greece

**Keywords:** artificial intelligence, machine learning, atherosclerosis, coronary artery disease, peripheral arterial disease, carotid artery disease

## Abstract

Atherosclerotic cardiovascular disease (ASCVD) is the most common cause of death globally. Increasing amounts of highly diverse ASCVD data are becoming available and artificial intelligence (AI) techniques now bear the promise of utilizing them to improve diagnosis, advance understanding of disease pathogenesis, enable outcome prediction, assist with clinical decision making and promote precision medicine approaches. Machine learning (ML) algorithms in particular, are already employed in cardiovascular imaging applications to facilitate automated disease detection and experts believe that ML will transform the field in the coming years. Current review first describes the key concepts of AI applications from a clinical standpoint. We then provide a focused overview of current AI applications in four main ASCVD domains: coronary artery disease (CAD), peripheral arterial disease (PAD), abdominal aortic aneurysm (AAA), and carotid artery disease. For each domain, applications are presented with refer to the primary imaging modality used [e.g., computed tomography (CT) or invasive angiography] and the key aim of the applied AI approaches, which include disease detection, phenotyping, outcome prediction, and assistance with clinical decision making. We conclude with the strengths and limitations of AI applications and provide future perspectives.

## 1. Introduction

**FIGURE 1:**
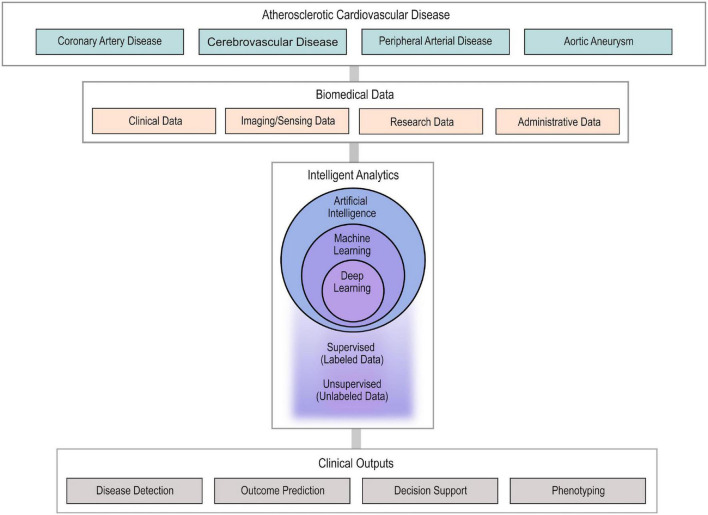
Usual workflow of AI approaches in ASCVD. Highly diverse biomedical data from patients with ASCVD are collected and used as input in AI algorithms to provide intelligent analytics. The developed AI algorithms (ML and DL) undergo supervised or unsupervised training to enable disease detection, outcome prediction, clinical decision support, and phenotyping. AI, artificial intelligence; ASCD, atherosclerotic cardiovascular disease; ML, machine learning; DL, deep learning.

Atherosclerotic cardiovascular disease (ASCVD) affects the coronary, cerebral and peripheral arteries and remains the most common cause of death globally (1). Over the last 60 years, numerous clinical trials, registries and prospective studies along with advances in basic science and biomedicine have formed our current understanding of the pathogenesis of ASCVD and have established the current clinical standards regarding diagnosis, prevention and treatment (2, 3). With the advent of digitalization and information era, large amounts of heterogenous ASCVD data, such as clinical, imaging, biosensing, administrative and basic science data, are now available (4). Big data analytics, artificial intelligence (AI) and particularly their computational branch of machine learning (ML) bear the promise of incorporating and utilizing these large amounts of heterogenous information to enhance our armament against ASCVD (5). Specifically, ML applications have already been employed, or are currently being researched, to improve ASCVD detection, assist with diagnosis, advance our understanding of ASCVD phenotypes in order to promote precision cardiovascular medicine, and to improve outcome prediction (Figure 1) (6–8). Experts in the field of ASCVD anticipate significant advancements and changes in the next 10 years (9). The broad spectrum of ML applications in ASCVD in combination with the lack of familiarity with ML methodologies for most cardiovascular physicians may create boundaries in the understanding and potential use of this technology in clinical practice.

Several reviews have been published to address this issue in ASCVD. In fact, the majority of existing reviews focus on coronary artery (CAD) and cardiac disease, in general (10, 11), or, more specifically, on imaging applications in CAD (12–14). For this reason, a separate category of reviews concentrates solely on non-cardiac ASCVD (15, 16). Also, complementary to the above-mentioned works, other studies provide more “technical” aspects by describing specific AI concepts and methods within the field of ASCVD (17–19). In our work, we attempt to provide not only a “clinical” aspect via an overview of AI applications in four forms of ASCVD (peripheral arterial disease—PAD, carotid artery disease—CAD, and aortic aneurysm—AA) but also “technical” insights about the specific AI concepts and algorithms. Thus, our focused overview of key AI concepts for the ASCVD specialist enriches the description of the use of AI in ASCVD clinical practice/research and the discussion about the future insights of AI as a tool to improve the personalized care in patients with ASCVD.

## 2. Key concepts

The term “big data” was coined to describe large quantities of information that is typically derived from different sources, in different formats and are produced at a high rate. AI is a broad term and refers to how human intelligence can be approached by artificial systems (20). The term includes theoretical approaches of what constitutes intelligence, as well as practical aspects such as computational methods. ML and pattern recognition constitute a computational branch of AI that aims to develop and apply algorithms for the purpose of recognizing patterns/regularities in data. ML algorithms require training, typically with the use of big data which are large and highly diverse datasets. For example, ML algorithms could be trained to recognize atherosclerotic plaques in cardiac computed tomography angiographies (CTAs). Training of such Machine learning algorithms would require supervision, so that the algorithm would be given previously labeled CTA images as an input, and its training would entail optimization of its parameters to a forced output (whether an atherosclerotic plaque is present or not). Accuracy of such ML algorithms would be then judged by their ability to correctly recognize the presence or absence of plaques in new CTA pictures. In another example, supervised ML may aim at predicting clinical outcomes. In this case, ML models are trained by forcing a known clinical outcome, e.g., long-term survival or death, to corresponding patient (e.g., cardiovascular) profiles (21).

Machine learning algorithms can be also trained without supervision (non-labeled data). Such an option is particularly helpful when the inherent structure of the available dataset, e.g., patient demographics/biometrics, medical images, or biosignals, is not known *a priori* (22). Moreover, there is often a need to perform phenotyping where groups of patients (clinical phenotypes) or images (imaging phenotypes) with similar characteristics are automatically identified within an initial population/dataset (23). ML can offer unparalleled ways for the identification of unknown phenotypes in “big data.” Most importantly, the identification of new, unknown phenotypes that are closer to individual patient characteristics, essentially promotes personalized medicine and its integration in everyday clinical practice (24).

The main representatives of commonly used ML algorithms include, but are not limited to neural networks, naive Bayes models, decision making trees, clustering algorithms, k nearest neighbor, and SVM. Deep learning (DL) essentially refers to neural networks with complex computational architectures and the use of additional layers of neurons to enhance performance (25, 26). We summarize key terms in Table 1, whereas Table 2 provides an overview of the ML applications in ASCVD described in the current work.

**TABLE 1:** Definitions of commonly used terms in artificial intelligence applications.

Term	Definition
Artificial intelligence (AI)	A broad term referring to how human intelligence can be approached and utilized by artificial systems.
Big data	Large quantities of digital data that are typically heterogenous and derived from different data sources and at high speed
Machine learning (ML)	A computational branch of AI referring to algorithms that perform intelligent tasks via recognition of patterns within Big Data. The term Pattern Recognition is essentially synonymous.
ML training	In the context of ML, it refers to the process of identifying the required parameters of a ML algorithm for a specific AI task via repetitive data analysis.
Supervised ML training	Training that is performed by forcing specific outputs to specific data.
Unsupervised ML training	Training that is performed without forcing specific outputs. In this case, ML algorithms create data patterns automatically, i.e., by utilizing specific mathematical criteria.
Artificial neural networks (ANN)	ML algorithms comprised of algorithmically interconnected nodes that resemble neural networks.
Deep learning (DL)	Refers to ANN that utilize complex, intermediate layers of nodes/neurons.

**TABLE 2:** Studies using machine-learning applications in ASCVD.

Study	Objective	Data type (number of patients or images)	ML algorithm	Supervised/unsupervised
**Disease detection/assisted diagnosis**				
Zreik et al. (50) 10.1109/TMI.2018.2883807	Detection and classification of coronary artery plaques	CCTA scans (163)	Convolutional recurrent neural network	Unsupervised feature extraction, supervised classification
Kang et al. (46) 10.1117/1.jmi.2.1.014003	Detection of non-obstructive and obstructive coronary plaques	CCTA scans (42)	Support vector machine	Supervised
Yoneyama et al. (47) 10.1186/s41824-019-0052-8	Detection of CAD	Hybrid SPECT/CCTA scans (59)	Artificial neural network	Supervised
Takx et al. (54) 10.1371/journal.pone.0091239	Automated CAC score classification	Non-gated CT scans (1,793)	Convolutional neural network pair	Unsupervised feature extraction, supervised classification
Wolterink et al. (55) 10.1016/j.media.2016.04.004	Automated CAC score classification	CT scans (250)	Convolutional neural network pair	Unsupervised feature extraction, supervised classification
Sandstedt et al. (56) 10.1007/s00330-019-06489-x	Automated CAC score classification	CT scans (315)	Not reported	Not reported
van Velzen et al. (57) 10.1148/radiol.2020191621	Automated CAC score classification	Non-contrast CT (7,240)	Deep learning network	Supervised
Tesche et al. (69) 10.1148/radiol.2018171291	Detection of lesion-specific ischemia based on non-invasive FFR	CCTA (85)	Deep learning framework	Supervised (FFR measured computationally)
Du et al. (72) 10.1016/j.jacc.2018.08.1360	Detection of stenosis	Invasive coronary angiography (5,050)	Deep learning convolutional neural network	Supervised
Cha et al. (77) 10.1038/s41598-020-77507-y	FFR via OCT-derived data	OCT data (141)	Random forest	Supervised
McCarthy et al. (104) 10.1002/clc.22939	Detect obstructive PAD	Clinical/biomarker data (354)	LASSO logistic regression	Supervised
Afzal et al. (106) 10.1016/j.jvs.2016.11.031	Detect PAD	EHR logs (1,569)	Natural language processing	Supervised
Kolossvary et al. (60) 10.1161/CIRCIMAGING.117.006843	Detection of napkin-ring sign (high-risk feature)	CCTA scans (69)	Logistic regression model	Supervised
Yin et al. (74) 10.3389/fcvm.2021.670502	Detection of plaque type (fibrofatty, calcified or lipid)	OCT images (2,000 images, 31 patients)	Convolutional neural network	Supervised
Chu et al. (75) 10.4244/EIJ-D-20-01355	Detection of plaque burden and plaque composition	CT scans (391)	Deep learning convolutional neural network	Supervised (manual annotation)
Oikonomou et al. (91) 10.1093/eurheartj/ehz592	Identify periarterial tissue inflammation	CCTA and biopsy-derived periarterial inflammation data (training-167, validation-44)	Random forest	Supervised
Qutrio Baloch et al. (111) (10.3390/diagnostics10080515)	Detect association between PAD severity and functional limitation	Clinical data from the STRIDES clinical program (703)	Ensemble strategy that compared random forest, neural networks and generalized linear model	Supervised
**Phenotyping/clustering**				
Oikonomou et al. (88)	Identify phenotypes that favor functional vs. anatomic evaluation of CAD	Clinical data from the PROMISE trial (9,572) -validated on SCOT-HEART trial	Extreme gradient boosting	Unsupervised
Yoon et al. (22) 10.1038/s41598-021-96616-w	Identify coronary plaque phenotypes	CCTA scans from PARADIGM (947)	K-means clustering	Unsupervised
**Clinical decision making**				
Buzaev et al. (101) 10.1016/j.cdtm.2016.09.007	PCI vs. CABG	Clinical/outcome data (4,679)	Artificial neural network	Supervised
**Outcome prediction**				
Le et al. (129) 10.1038/s41598-021-82760-w	Predict stroke based on carotid plaque features	Carotid CT scans (41)	Logistic regression model (ElasticNet)	Supervised
Christodoulou et al. (137) 10.1109/TMI.2003.815066	Predict stroke based on carotid plaque features	Carotid ultrasound images (230)	K-nearest neighbor	Supervised
Jiang et al. (122) 10.3389/fphy.2019.00235	Predict AAA expansion	Clinical data (20)	Deep belief network	Supervised
López-Linares et al. (140) 10.3389/fbioe.2019.00267	Predict EVAR outcome	Displacement Field-Based Strain (22)	Support vector machine	Supervised
Davis et al. (113) 10.1016/j.jvs.2016.11.053	Predict surgical site infection after lower extremity revascularization	Clinical data from the BMC2 VIC registry (3,033)	Ensemble strategy of 13 different techniques (Super Learner)	Supervised
Ambale-Venkatesh et al. (21) 10.1161/CIRCRESAHA.117.311312	Predict adverse cardiovascular events	MESA data (6,814)	Random forest	Supervised
Hathaway et al. (97) 10.1016/j.compbiomed.2021.104983	Predict adverse cardiovascular events	MESA data (6,814)	Deep neural survival network (DeepSurv)	Supervised
Kakadiaris et al. (7) 10.1161/JAHA.118.009476	Predict adverse cardiovascular events	MESA data (6,459)—validated on the FLEMENGHO study	Support vector machine	Supervised
Ward et al. (141) 10.1038/s41746-020-00331-1	Predict cardiovascular events in a multi-ethnic population	Outpatient EHR data (797,505 patients)	Ensemble strategy that compared gradient boosting machine, random forest, XGBoost	Supervised
Han et al. (98) 10.1161/JAHA.119.013958	Predict coronary atherosclerotic plaques at risk of rapid progression	CTCA scans from PARADIGM registry (1,083)	LogitBoost	Supervised
Bertsimas et al. (142) 10.1007/s10729-020-09522-4	Predict 10-year CAD-related adverse event	Clinical/outcome data (21,460)	ML voting system (ORT, CART, RF, LRM)	Supervised
Rao et al. (99) 10.1016/j.jcin.2013.04.016	Predict bleeding risk post-PCI	CathPCI registry (1,043,759 PCIs)	Binomial regression	Supervised
Motwani et al. (143) 10.1093/eurheartj/ehw188	Predict all-cause mortality in patients with suspected CAD	Clinical data from CONFIRM registry (8,844 patients)	LogitBoost	Supervised
van Rosendael et al. (144) 10.1016/j.jcct.2018.04.011	Predict CAD onset	Data from the CONFIRM registry study (8,844 patients)	XGBoost	Supervised
Kim et al. (145) 10.1016/j.jcmg.2018.04.009	Predict coronary atherosclerotic plaques at risk of rapid progression	CTCA variables from PARADIGM registry (1,083)	LogitBoost	Supervised
Motwani et al. (143) 10.1093/eurheartj/ehw188	Predict 5-year mortality in patients with suspected CAD	Clinical and CCTA variables (10,030)	Boosted ensemble algorithm	Supervised

ML, machine learning; CCTA, coronary computed tomography angiography; CAD, coronary artery disease; CAC, coronary artery calcium; SPECT, single positron emission computed tomography; FFR, fractional flow reserve; DL, deep learning; CNN, convolutional neural network; OCT, optical coherence tomography; RF, random forest; PAD, peripheral artery disease; LASSO, least absolute shrinkage and selection operator; EHR, electronic health records; LRM, logistic regression model; NRS, napkin ring sign; DCN, deconvolutional neural network; PVAT, perivascular adipose tissue; PCI, percutaneous coronary intervention; CABG, coronary artery bypass graft; CT, computed tomography; KNN, k-nearest neighbor; AAA, abdominal artery aneurysm; EVAR, endovascular aneurysm repair; GLM, generalized linear model; XGBoost, extreme gradient boosting; ORT, optimal regression tree; CART, classification and regression trees.

Regardless of the data or algorithms used, one can recognize four broad categories of clinical ML applications: (i) detection of disease, (ii) outcome prediction and risk assessment, (iii) phenotyping for better understanding of disease and precision medicine, and (iv) clinical decision support. Oftentimes, applications may serve more than one purpose, e.g., phenotyping may assist outcome prediction. In addition, many AI applications fall into the preclinical setting and are mostly the subject of engineers or data scientists who collaborate with ASCVD experts. In this work, we focus on AI applications that are mostly clinical or describe the clinical aspect of preclinical applications.

## 3. Coronary artery disease

### 3.1. Disease detection via medical recordings

Several studies have already focused on applying a wide variety of ML models to diagnose CAD using clinical recordings, or else patient demographics, symptoms and examination reports, electrocardiographic (EKG) and imaging data along with laboratory tests (27).

For example, Alizadehsani et al. employed different ML algorithms/methods, such as the Sequential Minimal Optimization (SMO), Naïve Bayes, Bagging SMO, and neural networks to diagnose CAD. The best predictive performance with an accuracy of 94.08% was achieved by the SMO models trained on the features selected using SVM weights (28). In another study, the same group proposed a hybrid ML method based on combining genetic algorithms to put forward the initial weights of the shallow neural network approach. The hybrid model outperformed the neural network model by 10% improvement in CAD diagnosis accuracy by achieving 93.85% (29). Similarly, Alizadehsani et al. applied the C4.5, Adaboost, SMO, and Naïve Bayes algorithms on more comprehensive recordings of laboratory and echocardiographic information. In this case, the SMO reported the best predictive capability with 82.16 ± 5.45% accuracy (30). Furthermore, Alizadehsani et al. explored the effect of adding a MetaCost algorithm to improve the efficacy of the ML models to detect/diagnose CAD. The authors implemented different ML models: Naïve Bayes, SMO, K-Nearest Neighbors (KNN), SVM, and C4.5. The study concluded that the combination of the SMO and MetaCost algorithms yielded very high sensitivity 97.22% and accuracy 92.09% in comparison to other models (31, 32). In another notable attempt by Nasarian et al. additional stress-, work-related and environmental features enriched an initial traditional clinical dataset in order to improve performance. The authors proposed a novel hybrid feature selection algorithm named heterogeneous hybrid feature selection (2HFS). The study trained different algorithms: Decision tree, Gaussian Naive Bayes, RF, and XGBoost classifiers. Outcome showed that the XGBoost classifier achieved a classification accuracy of 81.23% by employing the additional features (33). Moreover, Hassannataj Joloudari et al. proposed a novel hybrid ML model called “Genetic Support Vector Machine ANOVA” (GSVMA) method. The study combined a genetic optimization algorithm for feature selection and SVM with ANOVA kernel. The model achieved 89.45% accuracy in CAD prediction (34). Finally, Khozeimeh et al. employed cardiac magnetic resonance (CMR) image data for CAD detection. The newly developed ML model was based on a RF, trained on a new image features representation extracted from a convolutional neural network (CNN). The novel model achieved a 99.18% accuracy compared to the 93.91% of the CNN (35).

Apart from simply diagnosing CAD, several studies focused on detecting the location of stenosis in the coronary tree. For example, Alizadehsani et al. applied C4.5, Naïve Bayes, and KNN with the C4.5 model reporting the best performance for stenosis diagnosis by achieving 74.20 ± 5.51% for the LAD, 63.76 ± 9.73% for the LCX, and 68.33 ± 6.90% for the RCA (36). The later work was extended in Alizadehsani et al. (37), where the Bagging algorithm was employed for the same tasks and achieved 79.54, 61.46, and 68.96% for the LAD-, LCX-, and RCA-stenosis, respectively. Another study used SVM, which outperformed the reported results in the literature by achieving accuracy rates of 86.14% (LAD), 83.17% (LCX), and 83.50% (RCA), respectively (38). Alizadehsani et al. further elaborated on their previous work by training several SVM machines with different kernel methods: polynomial, linear, sigmoid, and radial basis functions to quantify the uncertainty in the RCA-, LCX-, and LAD-stenosis diagnosis based on the sample distance from the hyperplane. The SVM highest accuracies achieved were: 82.67±2.3% (linear kernel), 83.67±2.1% (sigmoid kernel), and 86.43±2.1% (linear kernel) for RCA, LCX, and LAD prediction, respectively (39). Recently, Alizadehsani et al. proposed a novel and improved feature selection method named “assurance feature selection.” Extracted features were again used to train SVM with different kernel methods achieving accuracies of 86.64% (LAD), 83.47% (LCX), and 82.85% (RCA) (40). In another approach, Alizadehsani et al. developed and proposed a two-stage ML model to improve the accuracy of CAD prediction. In the first stage, three classifiers were trained on weighted SVM extracted features to detect the stenosis in the three coronary arteries and a fourth classifier was built to predict/detect CAD cases. In the second stage, a new classifier was trained on the outcomes of the four first-stage classifiers to diagnose CAD. The proposed pipeline achieved a high accuracy rate of 96.40% in predicting CAD (41). Finally, Alizadehsani et al. introduced a comprehensive database to encapsulate all previous work in CAD diagnosis using ML techniques (42). Such an approach might facilitate the development of meta-databases and give a further boost to the use of ML in the management of future patients with CAD in order to improve prognosis and optimize outcomes.

### 3.2. Non-invasive imaging

#### 3.2.1. Automated plaque detection and coronary artery calcium calculation

Cardiac CTA is a widely used non-invasive modality for the diagnosis of coronary artery disease (CAD) (43, 44). Several studies have reported AI algorithms for the automatic detection of atherosclerotic plaques from CTA images (45). For example, Kang et al. developed a ML algorithm that identified coronary stenosis of 25% or more with an accuracy of 94% compared to visual identification of lesions with stenosis by expert readers using consensus reading (46). Yoneyama et al. developed a neural network for the detection of coronary stenoses from CTA images that cause perfusion defects on single-photon emission CT, achieving comparable results with physician experts (47). Several studies have reported neural networks that automatically grade CAD severity from CTA images according to the Coronary Artery Disease Reporting and Data System (CAD-RADS) (48–51), with satisfactory results (46, 47, 50). Such approaches could reduce the physician workload, time needed for diagnosis and the diagnostic accuracy (46, 49, 50, 52). Coronary artery calcium score (CACS) as assessed by computed tomography (CT) correlates with clinical ASCVD events and is used for risk stratification of asymptomatic individuals (51). CACS is typically calculated via a time-consuming semi-automatic methodology by physicians with the help of a software based on the Agatston score (51, 53).

Fully automated software assessing coronary calcification has been developed using AI algorithms with satisfactory reliability and agreement when compared to manual scores (54). Automated quantification of CACS can reduce the time of assessment without additional costs or exposure to radiation (55–57). In a recent study, van Velzen et al. developed and validated a DL method for automatic calcium scoring using different types of CT examinations that included the heart (e.g., diagnostic CT of the chest, PET attenuation correction CT, CAC scoring CT). The performance of the ML algorithm was evaluated against manual Agatson score and the results showed that the DL calcium scoring algorithm was robust, regardless of differences in CT protocol and subject population. Nevertheless, training of the algorithm with dedicated, protocol-specific images further augmented algorithm performance (57).

#### 3.2.2. Plaque characterization and detection of high-risk features

Machine learning algorithms have been developed to assist with atherosclerotic coronary plaque evaluation beyond the degree of stenosis. Masuda et al. for instance, developed a ML algorithm for identification of fatty vs. fibro-fatty atherosclerotic plaques from cardiac CTA with an area under the curve (AUC) of 0.92. ML could also be used to automatically identify high-risk features in coronary plaques, such as the napkin-ring sign (58). The latter identification has an additional importance, since the napkin-ring sign is a qualitative finding, thus automated identification could decrease inter-reader variability (59). Another interesting approach is ability of ML models to process hundreds of imaging features in order to identify known or discover new high-risk patterns in non-invasive studies. For example, a study showed that a large number of features are different between plaques with vs. without napkin-ring sign and exhibit remarkable discriminatory value, superior to conventional quantitative CT metrics (60).

#### 3.2.3. Derivation of functional indices

Machine learning can also assist with automatic evaluation of CT-derived functional indices such as fractional flow reserve (FFR) (61). FFR is a coronary physiology index that is considered the gold standard for ruling out obstructive CAD and assessing lesion severity of intermediate stenosis in patients with chronic CAD (62). FFR is derived during invasive coronary angiography with the insertion of a pressure-wire catheter, and it is defined as the ratio between maximal achievable blood flow in the presence of coronary artery stenosis and maximal blood flow in the absence of that stenosis. Notably, based on the results of several studies including the FFR vs. Angiography for Multivessel Evaluation (FAME I) trial, have suggested that an FFR below 0.8 corresponds to a hemodynamically significant stenosis that requires PCI, whereas an FFR above 0.8 suggests that optimal medical therapy is sufficient (63).

Several methods have been developed to estimate FFR based on computational flow dynamics (CFD) techniques applied to three-dimensional modeling of the coronary artery derived from angiogram or CT images without the use of invasive intracoronary wires. Example of software applications that have been developed and validated include Quantitative Flow Ratio (QFR), Vessel Fractional Flow Reserve (vFFR), and Fractional Flow Reserve Derived from Coronary Angiography (FFRangio). A number of studies have also developed methods to derive CT-FFR based on CFD techniques, and CT-FFR has been reported to have good diagnostic accuracy, prognostic value and therefore potential for clinical utility. Nevertheless, commercially available CFD software platforms to derive angiography-based or CT-based FFR have been restricted due to their low availability as they require remote and computationally demanding analyses significantly increasing the cost and delaying the diagnostic process (64–66).

Thus, ML algorithms have been used as an alternative to calculate FFR with less computational requirements. ML models have been trained using geometrical and flow data from 3D coronary models derived from coronary angiograms or CT images. Importantly, ML-based software can be used on-site by standard configuration personal computers (67, 68). Preliminary results show that ML-based CT-FFR performs equally well compared to CFD-based CT-FFR at decreased cost (69, 70). To illustrate, a non-commercial software that uses DL to evaluate CT-FFR has been developed and tested by Rother et al. Compared to invasive FFR, CT-FFR showed high accuracy in detecting ischemia and a significant reduction in calculation time when compared to existing CFD models that calculate FFR from CT images. More recently, the accuracy of the software was also tested using previous generation CT scanners to assess whether the imaging quality could potentially affect the results (71). However, the results showed good diagnostic performance for detection of flow-limiting obstructive coronary lesions. ML-based software that estimate FFR can enable a wider implementation of this promising coronary physiology index that has proven to be an important indicator of coronary ischemia and predictor of adverse coronary events.

### 3.3. Invasive imaging

#### 3.3.1. Automated lesion detection and characterization

Similar to non-invasive coronary imaging, ML algorithms can enhance the efficacy of invasive coronary angiography by enabling the automated detection of stenoses and their types, as well as the conduction of quantitative coronary angiography (QCA). For example, Du et al. trained a deep neural network using over 6,000 coronary angiograms to automatically identify stenoses and characterize them as dissections, thrombotic or calcified lesions (72). Apart from assistance in detection of disease, ML algorithms could also assist with quantitative angiography, which removes the subjective interpretation of lesions, however, is not widely used today. A significant number of researchers have focused on applying ML algorithms to intravascular imaging such as intravascular ultrasound and optical coherence tomography (OCT), which are used today complementary to angiography for plaque characterization and importantly, optimization of percutaneous coronary intervention (PCI). Specifically for OCT, ML algorithms can achieve high accuracy in identifying lesion composition (73–75). Recently, the FDA approved Abbotts’ Ultreon™ 1.0 Software for automated and assisted OCT use (Abbott, Abbott Park, IL) (76). ML algorithms are also being investigated to further improve functional indices of coronary lesions, such as FFR, or calculate them via alternative means. For example, a ML algorithm was developed to measure FFR from OCT-derived data showing high correlation (*r* = 0.85, *P < 0.001*) with invasive FFR (77).

#### 3.3.2. Assistance with clinical decision-making regarding revascularization

The incorporation of angiographic data and functional indices could be used to offer an overall assessment of a coronary lesion with regards to the decision for revascularization or not, essentially mimicking human expertise. CEREBRIA-1 study was a representative study multinational study (ML vs. Expert Human Opinion to Determine Physiologically Optimized Coronary Revascularization Strategies), where DL was used to approach the clinical decision making of world experts in this matter (78). The study aimed to evaluate the predictive accuracy of a ML model developed based on computational interpretation of pressure wire pull back data. The ML algorithm was compared to expert human interpretation in determining the need for PCI as well as for the decision of PCI strategy in patients with stable CAD. The results showed that the ML program was non-inferior to expert opinion. The notable benefit of this approach is that the ML algorithm essentially could provide an expert recommendation to the interventional cardiologist of every catheterization laboratory.

#### 3.3.3. Image quality improvement via ML

In addition to automated lesion characterization and clinical decision-making regarding revascularization, AI may further assist the interventional cardiologist by detecting artifacts or poor image quality and correct it appropriately and automatically (79). Such an option might have essential clinical impact, by improving the accuracy of calculated functional indices, which frequently drive decision making, especially taking into account that almost 30% of the functional indices derived intraoperatively in catheterization laboratories cannot be reliably used for clinical decision-making (80).

### 3.4. Assisted diagnosis of myocardial infarction

Physicians are extremely capable in diagnosing and treating myocardial infarction (MI) in symptomatic patients seeking medical care. A systematic review has reported low rates of missed MIs in the hospital setting, ranging between 1 and 2% (81). Hospitals in rural areas and those with a low proportion of classical chest pain patients were at greater risk for missing an MI. ML models to enhance automated detection of MIs from 12-lead EKGs have been developed with accuracy comparable to that of cardiologist (77, 82). EKG machines equipped with this ML software could therefore decrease the rates of in-hospital missed MIs even further or could help emergency medical services enhance their response times in patients with MIs. The most significant contribution of ML technology, however, would be the detection of MIs and their complications in the community.

In patients suffering from ST elevation MI (STEMI), delay to reperfusion translates into more extensive myocardial injury and increased mortality: every minute counts and hospital systems have adopted internationally accepted response times referred to as door to balloon time (83). However, a critically important time is frequently lost before patients seek medical care. Smartwatch technology is already being used for the screening and detection of atrial fibrillation in the community setting (84), and an expansion of its use for the detection of MIs is very appealing. However, the problem is more complex, as atrial fibrillation screening can be performed with analysis of pulse or a single EKG lead, whereas EKG detection of MI requires multiple EKG leads. Spaccarotella et al. showed in a pilot study that a 9-lead EKG obtained asynchronously from a single smart watch device could be used to detect STEMI and non-STEMI with good accuracy compared to a standard 12-lead EKG (85). This technology could be coupled with ML algorithms to enable the early detection of MIs in the outpatient setting, and in fact, studies toward that goal are already being conducted (86). Importantly, as in the case of atrial fibrillation, prospective studies will be required to prove the clinical validity and benefit of such new technologies.

### 3.5. Phenotyping and precision medicine

Machine learning methodologies have been applied in CAD datasets with the purpose of discovering new clinical phenotypes that can improve our understanding of the disease and/or our ability to better predict outcomes. In general, as the number of phenotypes increases, prediction comes closer to the individual level and ML facilitates precision medicine. However, there is no strict definition as to what constitutes precision medicine.

A classic example of imaging phenotyping in CAD is a ML algorithm developed by Yoon et al. where unsupervised training led to the creation of four different groups of plaques based on their imaging characteristics from the PARADIGM registry (22). Patients from cluster 1 had plaques that consisted of a necrotic core surrounded by mainly fibrotic and lipid tissue. In cluster 2, plaques were mainly fibrotic and fatty, in cluster 3, the plaques had mainly fibrotic and calcified tissue, and in cluster 4 plaques were mostly calcified. Patients with plaques classified to clusters 2 and 3 had the highest risk for major adverse cardiovascular events, whereas cluster 1 had the lowest risk. These results can assist our understanding of what constitutes a high-risk plaque and how it can be used to predict outcomes. Furthermore, ML models have the ability to process hundreds of imaging features and discover new important characteristics (87).

A characteristic example of clinical phenotyping and precision medicine in CAD was recently published by Puchner et al. (59), where a phenomapping-derived tool was developed and validated with the aim to personalize the selection of anatomical (cardiac CT) vs. functional testing (stress testing) in individuals with suspected CAD (88). The investigators included participants from PROMISE trial (ClinicalTrials.gov identifier: NCT01174550) where individuals presenting with stable chest pain were randomized to either anatomical (CTCA) or functional testing (exercise electrocardiography, nuclear stress testing, or stress echocardiography) (89). Utilizing baseline characteristics including, among others, demographics, anthropometrics, cardiovascular risk factors, and laboratory measurements, the investigators first broke down the trial cohort to numerous, distinct phenotypes based on a data similarity index. For each phenotype, they then estimated the hazard ratio of major adverse cardiac events with stress test vs. cardiac CTA. Training of an extreme gradient boosting algorithm was then performed to identify patient features that were strongly associated with improved outcomes. Feature performance was evaluated using SHAP (Shapley Additive Explanations) values which identify a predictor contribution, either positively or negatively, to the prediction. Finally, internal validation of the algorithm was performed in a subset of patients from PROMISE trial, while external validation of the chosen method was also performed using the Scottish Computed Tomography of the Heart (SCOT-HEART) trial (ClinicalTrials.gov identifier: NCT01149590) and showed a reduction in the composite clinical endpoint (88, 90).

In another study by the same group, researchers developed a ML algorithm based on > 1,000 cardiac CTA perivascular adipose tissue features to predict the probability of major adverse cardiac events within 5 years in patients with stable CAD, defining it as the “fat radiomic profile” (91). They further examined the correlation of CTA features with genes expressing inflammation, fibrosis, and vascularity. A “fat radiomic profile” above 0.63 was linked with a 10-fold increase in the risk of MACE, even after adjustment for all pertinent covariates. In the same study, the authors point out that lack of standardization in coronary CTs and image post-processing remains a problem that can hinder the robustness of these methods—namely, the ability to obtain comparable CTA features results from a wide variety of hardware, scan settings and software configurations (91).

### 3.6. Risk assessment and outcome prediction

Risk assessment is important for different subgroups of CAD patients and guides patient management. In people without known CAD, several tools have been developed and are currently used for the risk assessment and prediction of future cardiovascular events, including the Framingham score, SCORE and SCORE2, as well as the American College of Cardiology/American Heart Association (ACC/AHA) risk tool (92, 93). These tools were derived using traditional statistical methods and show only moderate to good discrimination in predicting ASCVD outcomes (94–96).

ML models have been developed with the goal of improving the predictive accuracy of these tools. In a recent study, Ambale-Venkatesh et al. combined deep phenotyping with ML to train an algorithm for cardiovascular disease prediction (21). Using over 700 clinical, imaging, laboratory, and biomarker variables from the Multi-Ethnic Study of Atherosclerosis (MESA) study (2), a prospective study of over 6,000 asymptomatic individuals with serial ASCVD evaluation and long term follow up, the authors developed a ML algorithm to predict cardiovascular outcomes, including all-cause mortality, coronary heart disease, and stroke, over 12 years of follow-up. Specifically, a random survival forests ML technique was used to identify the 20 most important predictors of cardiovascular outcomes and the final ML model achieved better predictive accuracy compared to established risk scores (21). Results from a similar ML algorithm using data from MESA also showed improved predictive accuracy (97). As an example of the clinical importance of improved predictive accuracy achieved by ML algorithms, application of an ML model developed based on data from the MESA and the Flemish Study of Environment, Genes and Health Outcomes (FLEMENGHO) would have reduced statin prescriptions by 68% while simultaneously increasing the statin use of true high-risk patients (patients that went on to experience a CVD event) by 52% (7). Besides improving prognostication, ML analyses can improve our understanding of ASCVD by showing the important role of biomarkers; tissue necrosis factor-alpha, C-reactive protein, fibrinogen, interleukin-2, and interleukin-6 were top predictors of outcomes in the abovementioned ML models. In contrast, established risk assessment tools do not incorporate such biomarkers.

In patients with known or suspected CAD, estimation of CAD progression can also be clinically helpful. Along these lines, an ML model developed using clinical and imagine data of patients that underwent serial cardiac CTA from the PARADIGM study, demonstrated reasonably good predictive value for progression of coronary plaque (area under the curve 0.83) (98).

In patients with CAD undergoing PCI, assessment of bleeding risk remains challenging. The American College of Cardiology has developed a bleeding risk score using data from over 1 million PCIs from the CathPCI registry (99). Although ML techniques have not proven to outperform the CathPCI risk score in a head-to-head comparison, results of a ML study have been promising (100).

Lastly, ML-based models may in the future have a role in clinical decision-making beyond risk assessment, as in assisting with revascularization strategy (101).

## 4. Peripheral arterial disease (PAD)

### 4.1. Detection of disease

Peripheral arterial disease refers to the narrowing of the arteries that supply the upper and lower limbs, most commonly due to atherosclerosis (15). Although it is estimated that more than 200 million patients worldwide suffer from PAD (102), more than 50% of these patients are asymptomatic and often remain undiagnosed and untreated (103). Advanced tools, such as AI, have been used for facilitating not only the detection, but also the management and outcome prediction in PAD (15).

For example, an ML algorithm, which employs clinical variables (e.g., history of hypertension) and serum biomarkers, was used to create a unique score and predict the presence of PAD in a sub-population of CASABLANCA registry’s including patients referred for diagnostic peripheral and/or coronary angiography (104). The novel score achieved a sensitivity of only 65% but a specificity of 88% with a positive predictive value of 76% with the cut-off having been optimally set (104). In another study targeting elderly patients, a random forest (RF) approach provided better results in diagnosing PAD compared to simple logistic regression and the traditional ankle-brachial index (ABI) (105). In fact, the seven most important features of the developed RF model were the: (i) ABI, (ii) creatinine level in blood, (iii) fasting blood glucose, (iv) age, (v) presence of CAD, (vi) presence of diabetes, and (vii) presence of hypertension.

In another approach the clinical narrative notes of 1,569 patients were processed using an AI natural language processing (NLP) algorithm (106). Results were compared to algorithms employing the International Classification of Diseases (ICD) codes, as well as the ABI measurements of the patients. The NLP outperformed its competitors (*P < 0.001*) yielding 91.8% accuracy, 92.5% sensitivity, and 92.9% positive predictive value (106). In a follow-up study, the same team assessed the efficacy of the NLP approach to diagnose critical ischemia of lower extremities (107). As previously, they compared the knowledge-driven NLP algorithm to an ICD-based one. Despite having no statistically significant difference in specificity, the NLP method had better positive predictive value, specificity and F1-scores (*P < 0.001*) compared to the ICD model (107), indicating the superiority of AI-based NLP in diagnosing PAD, compared to other traditional approaches. Moreover, Weissler et al. performed a comparison between the NLP of medical notes and the least absolute shrinkage and selection operator (LASSO) ML methodology, which is based on administrative data (108). The NLP algorithm led to an AUC of 0.888, whereas the LASSO’s AUC was 0.801 (*P < 0.0001* according to DeLong test) (108). Abovementioned studies highlight the capability of NLP in PAD detection with great implications for initial PAD screening in everyday clinical practice.

Furthermore, Ghanzouri et al. used electronic health records (EHRs) to detect undiagnosed PAD in a cohort of 3,168 patients. The authors compared the developed DL-based approach to traditional models showing a clear superiority of the DL model (average AUC of 0.96) over RF (average AUC of 0.91), and logistic regression approaches (average AUC of 0.8). Another significant point of that study is the demonstration, via a corresponding analysis, that clinicians are generally receptive to automated EHR-based models for PAD detection (109).

Moreover, apart from clinical and EHR data, AI may well be applied on other data types to achieve PAD detection. McBane et al. applied DL for the detection of PAD via arterial (posterior tibial artery) Doppler waveform data. In specific, the proposed DL algorithm predicted normal (>0.9) or pathological (≤0.9) post-exercise ABI based on posterior tibial artery Doppler waveforms recorded at the resting state. The model included 1,941 patients with PAD and 1,491 control subjects and detected PAD with an AUC of 0.94 (CI = 0.92–0.96) (110).

The heterogeneity of the datasets included in the above-described studies demonstrates the importance of employing diverse datasets in detecting PAD.

### 4.2. Severity stratification and outcome prediction

The option to accurately assess disease severity facilitates not only the patient management process, but also the objectification of applied therapies. Qutrio Baloch et al. investigated the relationship between disease severity and patient functional impairment (111). In total, administrative data from 703 patients were analyzed with supervised ML. Quality of life, 6-min walk test scores, calf circumference, toe-branchial index, and basic demographics constituted the feature set (111). Despite the not striking results of the applied ML method, compared to conventional statistical methods, the study highlighted the non-linear relationship between disease severity and mobility restriction (111), showing that AI interventions provide results if not better at least as accurate as conventional statistics do.

In another study, Sonnenschein et al. developed a ML approach with the use of a multi-dimensional set of clinical features (demographics, biometrics, blood tests) to calculate an AI-based PAD score (AI-PAD). The calculated score (range 0–100) categorized the patients with PAD (*n* = 46) into two groups (stable PAD, sPAD, Fontaine stages I–II and unstable PAD, unPAD, Fontaine stages III–IV) based on a cut-off value of 50 AI-PAD units (AI-PAD < 50 for sPAD and AI-PAD > 50 for unPAD) and showed good correlation with the measured ABI values and severity of disease (112).

Outcome prediction in PAD is a pivotal step to be taken after diagnosis, as it sets the strategy for the patient management to be followed. A study conducted by Davis et al. aimed to reveal predictive indices of infection of the surgical wounds in patients undergoing open surgical revascularization (via bypass) of the lower limbs (113). The authors utilized an ML technique (Super Learner algorithm) to develop a prediction model for surgical wound infection. The study population included 3,033 patients in total. Major predictors of postoperative infection were dialysis-requiring renal failure (OR 4.35, *P* < 0.001) and hypertension (OR 4.29 *P* < 0.001) (113).

In another study, 81.930 patients were analyzed using the LASSO ML methodology to select variables and define a score (OAC^3^-PAD Risk Score) which can effectively predict the major bleeding events after the hospitalization for PAD (114). Independent predictors were oral anticoagulation therapy, age > 80, presence of chronic limb threatening ischemia, congestive heart failure and severe chronic kidney disease, previous bleeding event, anemia, and dementia. The novel score displayed good calibration and discrimination among four risk groups (*c* = 0.69, 95% confidence interval 0.67–0.71) ranging from low (1.3%) to high bleeding risk (6.4%) in the first year after the hospitalization for PAD.

Finally, Ross et al. developed several tailored ML models which included clinical, demographic, imaging and genomic data to predict future mortality in patients with PAD (115). The newly designed ML models outperformed (AUC 0.76 vs. 0.65, respectively, *P* = 0.1) traditional approaches (logistic regression models) for the task-at-hand: the prediction of future mortality. The studies described above demonstrate the wide range of applicability for AI in the management of patients suffering from PAD.

## 5. Abdominal aortic aneurysm

Abdominal aortic aneurism (AAA) is one of the most severe complications of ASCVD conveying significant mortality risk due to rupture (116). Its size and growth rate determine the risk of rupture and, thus, the necessity for operative therapy (116). AI has been already employed in the management of AAA, mainly in setting the diagnosis, predicting its growth and risk of rupture or in the preoperative planning and postoperative outcome prediction (117).

Computed tomography angiography is the gold-standard technique for the diagnosis, as well, as the preoperative planning and postoperative outcome assessment in AAA. For this reason, the majority of AI techniques in AAA management are CTA-based. Adam et al. employed a DL approach (Augmented Radiology for Vascular Aneurysm—ARVA) to detect an AAA and measure its maximal diameter in 489 CTA scans (118). ARVA outcomes were compared to a reference expert, demonstrating a median absolute difference of 1.2 mm, while the median absolute differences of another six experts compared to the same reference expert were 1–2 mm. Lareyre at al. combined a supervised DL algorithm with a feature-based expert system to improve the accuracy of the automatic segmentation of the abdominal aorta and its major branches (119). Such AI-powered approaches could facilitate preoperative planning of endovascular AAA interventions, especially in complex anatomies.

In another application, the prediction of AAA growth, Hirata et al. used as an input to an ML algorithm 9 CTA-extracted anatomic features of small AAAs (38.5 ± 6.2 mm) (120). ML achieved an AUC of 0.86 in predicting expansions of more than 4 mm per year outperforming traditional features, such as the AAA major axis (AUC of 0.78) (120). Along the same lines, Kontopodis et al. developed an AI-based approach that could stratify AAAs into high and low growth rate groups. Using a diverse set of 29 different variables (clinical, biological, morphometric, and biomechanical), a gradient boosting (XGboost) and a support vector machines (SVM) model were trained in order to predict which AAA would reach a growth rate higher than the cohort median. XGboost achieved the highest AUC 81.2% in predicting high growth rate AAAs compared to low growth rate ones. The study included a small cohort of 40 patients with small AAAs (maximum diameter 32–53 mm) (121).

Nevertheless, in order to precisely predict the growth of AAAs, usually larger longitudinal datasets are required. Jiang et al. tried to tackle the usual problem of lack of such datasets by employing a two-step computational approach to generate an expanded *in silico* dataset of AAA growth and structural features (122). Then, they employed a DL algorithm to combine both *in silico* and real CTA patient data to predict the evolution of the AAA in 20 patients. The DL method outperformed a conventional mixed-effect model by 65% in predicting the size increase of AAAs, showcasing an average relative error of 3.1%.

The issue of the need for large datasets in order to apply AI in the management of AAA is also discussed in a study by Fujiwara et al. where the authors evaluate the accuracy of AI to detect and measure AAA using limited-size CT datasets. The authors employ label-free CTs, avoiding possible complications associated with the intravenous use of contrast, such as renal failure or allergic reactions. In a dataset of 145 label-free CT scans (*n* = 111 with AAA), the proposed approach achieved a sensitivity of 94.6% for AAA detection and a good estimation of the AAA size (42.5 ± 8.8 mm) compared to those of diagnostic reports (44.6 ± 8.4 mm) (123).

Even if endovascular aortic repair (EVAR) is generally characterized by good outcomes (124), it can be still be followed by complications, such as the endoleak (EL). Korzadeh et al. applied AI to predict not only the presence, but also the severity of EL (I–III). The model was fed with 26 non-imaging clinical attributes (e.g., biometrics, demographics, blood values) recorded preoperatively and achieved an overall accuracy of more than 86% (125). The authors notice that the model may well be further enhanced with imaging data, highlighting the adaptability of AI in different and diverse datasets and its high potential to provide even more powerful tools for the clinical management of AAA in the future.

Finally, in an attempt to provide insights into the risk for rupture—the most serious complication of AAA, Chung et al. used AI to predict wall stress in AAAs: a feature that is associated with the risk for rupture. The novel AI-based framework was compared to traditional analysis in terms of AAA automatic segmentation, 3D geometry reconstruction and aortic wall stress calculation. The trained U-NET was found to perform in a statistically similar way compared to traditional analysis but in a significantly smaller amount of time (20 s vs. 4 h) (126).

Although the role of AI in the management of AAA seems promising, the aforementioned results are still preliminary and remain to be evaluated in large-scale clinical trials.

## 6. Carotid artery disease

Imaging plays a pivotal role in the management of carotid artery disease. For example, CTA is frequently performed to set the diagnosis and plan the surgical treatment of a patient (e.g., open vs. endovascular). The decision to operate is typically based on the degree of carotid stenosis and the presence of symptoms. However, patients often have transient ischemic attacks or stroke with only mild to moderate carotid luminal stenosis (127). Moreover, studies show that a more detailed plaque characterization expanding beyond luminal stenosis could provide additional value in predicting future events (128).

In a recent study, radiomic features were extracted from carotid CT scans of patients with cerebrovascular events to investigate their robustness and reliability, and whether they could provide incremental prognostic value in identifying high-risk culprit carotid arteries from non-culprit carotid arteries (129). Using feature extraction hyperparameters, the researchers ended up with 93 radiomic-derived variables, more than half of whom displayed high robustness to simulated inter-observer variability in region of interest (ROI) demarcation. Using only the top 10 informative and robust radiomics variables, the authors trained an ElasticNet logistic regression model that outperformed the calcium score alone in pinpointing culprit lesions. When both modalities were combined, an AUC of 0.73 was achieved (129).

Radiomic features have also been extracted from carotid MRI scans to construct a high-risk plaque model for differentiating symptomatic from asymptomatic carotid plaques (130). In a recent study by Zhang et al. 162 patients with carotid stenosis were randomly divided into training and test cohorts. Multi-contrast and contrast enhanced MRI images were obtained and radiological features of carotid plaques were recorded to build a traditional model. Additionally, radiomic features on these images were derived to construct a high-risk MRI based model with least absolute shrinkage and selection operation algorithm in the training cohort. The performance of the model was evaluated in the test cohort. Finally, a combined model was developed using both the traditional and the radiomics model and a comparison between the traditional, the radiomics and the combined models was performed. The radiomics model could accurately distinguish symptomatic from asymptomatic carotid stenosis and was found to be superior to the traditional model in the differentiation of high-risk plaques while the combined model did not provide any additional benefit compared to the radiomics model (130).

Carotid plaque morphology as recorded by high-resolution ultrasound images can also have prognostic implications and assist in identifying asymptomatic individuals with carotid stenosis at risk for stroke (131). Stable plaques are assumed to present as echogenic, smooth, and homogeneous, while vulnerable plaques typically are echolucent, irregular, and heterogeneous (132, 133). Kordzadeh et al. applied AI in the detection of carotid artery disease via static grayscale duplex ultrasound images. A dataset of 156 ultrasound images (with and without carotid artery stenosis) was used to train a geometry group network based on CNN architecture. The algorithm detected carotid artery stenosis of any grade with a sensitivity, specificity, and accuracy of 87, 82, and 90%, respectively (134). Jain et al. used an Attention-UNet DL model to identify/segment carotid plaques in complex ultrasound images with bright and fuzzy plaques of the internal (ICA) and common carotid artery (CCA). The study included 970 ICA and 679 CCA images from three different centers. The performance of Attention-UNet model was benchmarked against UNet, UNet++, and UNet3P models yielding an AUC value of 0.97, compared to 0.964, 0.966, and 0.965 AUC values for the three other models, respectively (135). Along similar lines, Latha et al. classified 361 carotid ultrasound images into normal or pathologic by means of several different ML methods, such as the CART decision tree, RF, logistic regression, CNN, Mobilenet, and Capsulenet. The latter was characterized by a superior classification performance, as reported by a 12.91, 8.33, 5.47, 43.12, and 1.75% improvement in accuracy compared to the CART decision tree, logistic regression, RF, CNN, and Mobilenet, respectively (136). Finally, in another study, a computer-aided system could provide a more standardized and accurate classification of carotid plaques. As an early example of such an approach, a computer-aided classification system was developed using multi-feature texture analysis, neural network classifiers, and statistical pattern recognition techniques. The system managed to automatically characterize carotid plaques imaged with high-resolution ultrasound, achieving an AUC of 0.75 at predicting which patients would develop stroke (137).

Studies described above demonstrate how AI can facilitate the management of carotid artery disease by providing effective and automated image-based tissue characterization and patient classification.

## 7. Challenges and limitations in the application of AI in ASCVD

Although the applications of AI in ASCVD are promising, several challenges and limitations remain. First, overtraining may lead to overfitting of the applied ML model and, thus, poor generalization with limited performance using real-world data. This challenge can be overcome by using large, trustworthy datasets that are representative of the target population as well as appropriate ML algorithms. Second, in contrast to classic statistical approaches, many ML implementations are characterized as “black-box” or else “non-explainable,” because the patterns that are created cannot be easily interpreted. As a result, ML models cannot be easily trusted by expert users, which is particularly true for ASCVD physicians. The development of explainable AI (xAI) approaches is an answer to this limitation. For example, SHAP (Sharpley Additive Explanations) explainability analysis approximates any complex, generalized ML prediction model with local, linear models that explain individual predictions using a game theory approach (138). Third, AI requires the collection and pre-processing of large and diverse datasets, which can be time-consuming. The development of international database collaborations for the availability of data to researchers globally could overcome some of these challenges (139). Fourth, the development and application of AI in ASCVD requires not only high computational power but also specialized AI skills and steady collaboration between data scientists and physicians. To this end, tailored AI frameworks and ASCVD-AI laboratories are under development. Finally, although ML algorithms are clearly superior in automated detection of disease and phenotyping, one has to recognize that results in risk assessment and prognostication have often been inferior to traditional statistics.

## 8. Conclusion and future directions

**FIGURE 2:**
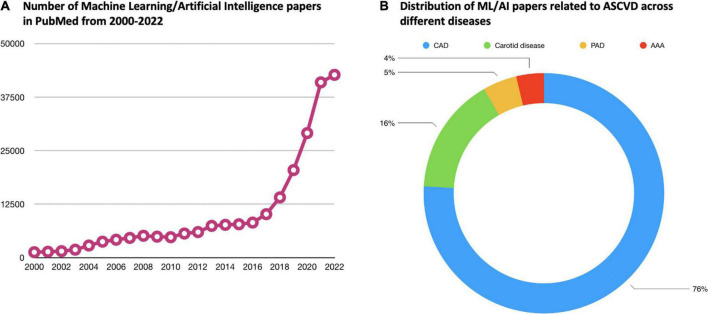
Current body of evidence related to ML/AI in the field of ASCVD. **(A)** Demonstrates how the number of papers in PubMed related to ML/AI has increased dramatically over the past two decades. **(B)** Shows how the ML/AI papers related to ASCVD specifically are distributed across the different diseases under the umbrella of ASCVD. In PubMed, the vast majority of ASCVD ML/AI papers are related to CAD. ML, machine learning; AI, atherosclerosis artificial intelligence; ASCVD, atherosclerotic cardiovascular disease; CAD, coronary artery disease; PAD, peripheral artery disease; AAA, abdominal aortic aneurysm.

As presented herein, AI methodologies, in combination with the growing availability of “big data” have already started enhancing disease detection, risk assessment, phenotyping, and providing clinical decision support in ASCVD. Over the past two decades, there is a growing interest in ML and AI especially in the field of ASCVD (Figure 2). Applications empowered by AI algorithms are becoming daily practice, particularly in the field of cardiovascular imaging. For this reason, there is a significant interest for the development of community-based ML applications that can be embedded in smart portable or wearable devices for different purposes (e.g., detection of acute coronary syndromes). Researchers are now applying ML algorithms to uncover previously unknown or neglected associations, such as the role of image- or signal-based biomarkers in predicting cardiovascular events. In parallel, the application of ML in large datasets bears the promise of leading to a more precision medicine approach in the risk assessment and therapy of patients with ASCVD.

However, current ML models in the ASCVD field are still in their infancy, focusing mainly on the classification and regression models for a single task-at-hand. Nevertheless, available AI models can go beyond those basic tasks, for example toward generating more realistic medical images using generative models such as Variational Auto-Encoders (VAEs) and Generative Adversarial Networks (GANs). One of the major challenges in the medical domain is the limited number and size of patient datasets for training ML or AI models. A promising AI field called meta-learning aims at solving this problem by building models that can learn from limited-sized datasets with only few data points. Several algorithms are listed in this domain, so far, such as: Siamese Networks, Prototypical Networks, and Relation Networks. Finally, another trending AL approach is deep multimodal learning that focuses on linking patient information such as EHRs, genomics, and imaging with multiple techniques to improve the final regression/classification performances.

As different needs and applications arise in the ASCVD field, an even more disseminated use of AI approaches in managing and studying ASCVD is expected to be established in the near future. On the one hand, the ASCVD physician should seek a stronger and stable collaboration with AI experts in order to increase familiarity and understanding with this exciting technology. On the other hand, the AI expert should focus on tailoring developed approaches to real-world ASCVD physician needs. In any case, the future ASCVD patient is going to benefit from a more effective, precise, and personalized management of disease in daily practice.

## Author contributions

PK: design, synthesis, and final editing. ME, N-AF, CB, DM, AT, VV, MK, and H-HE: synthesis. LH and AA: synthesis and editing. AK: synthesis and final editing. All authors contributed to the article and approved the submitted version.
